# Supplementary figures and images for: Pu.1/Spi1 dosage controls the turnover and maintenance of microglia in zebrafish and mammals
Source: eLife. 2025 Jul 17;14:RP105788. doi: 10.7554/eLife.105788 (PMC12270482; doi:10.7554/eLife.105788)

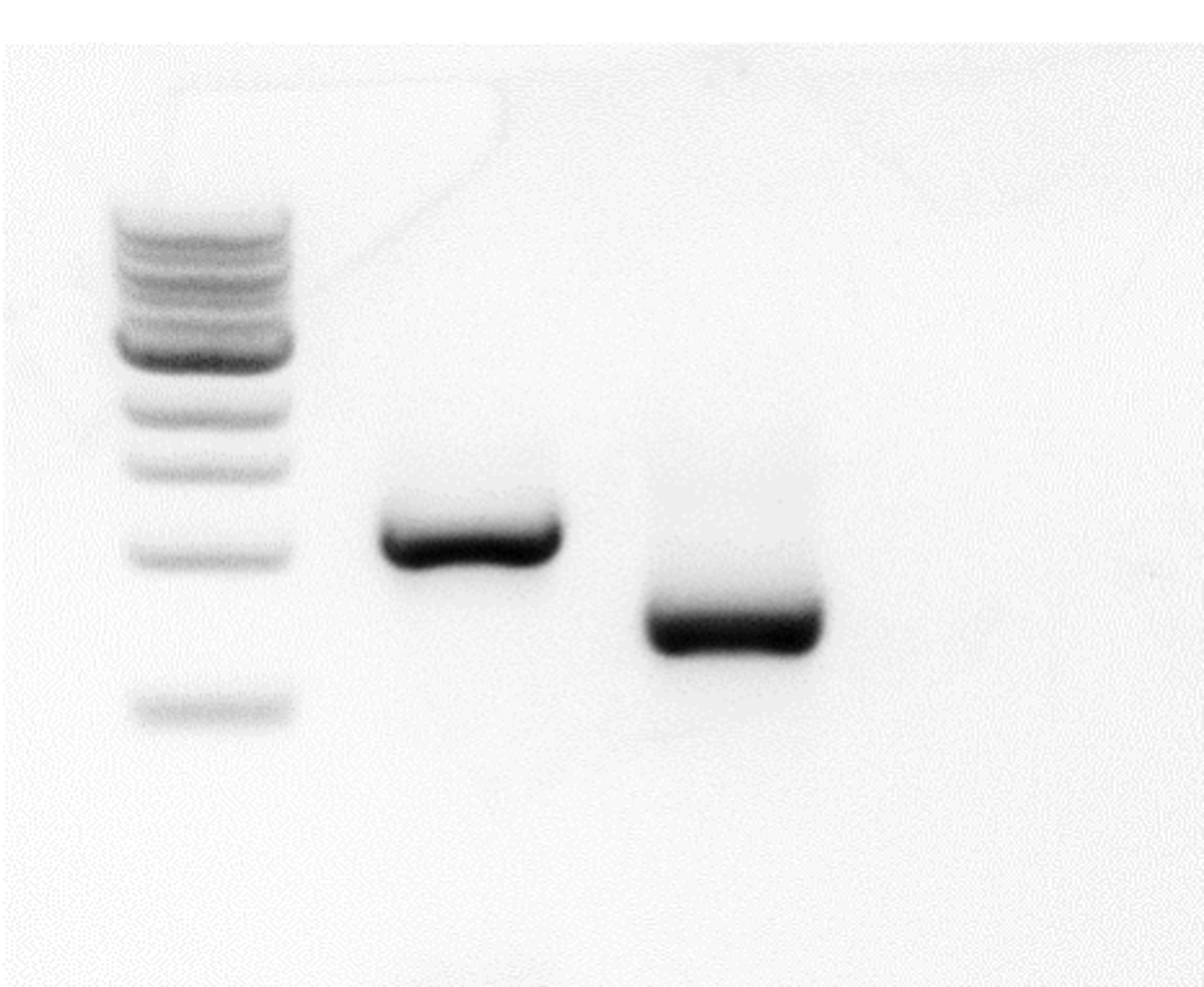

Supplement: Figure 2—figure supplement 1—source data 1. [file elife-105788-fig2-figsupp1-data1.zip › Figure 2-figure supplement 1-source data 1.tif]

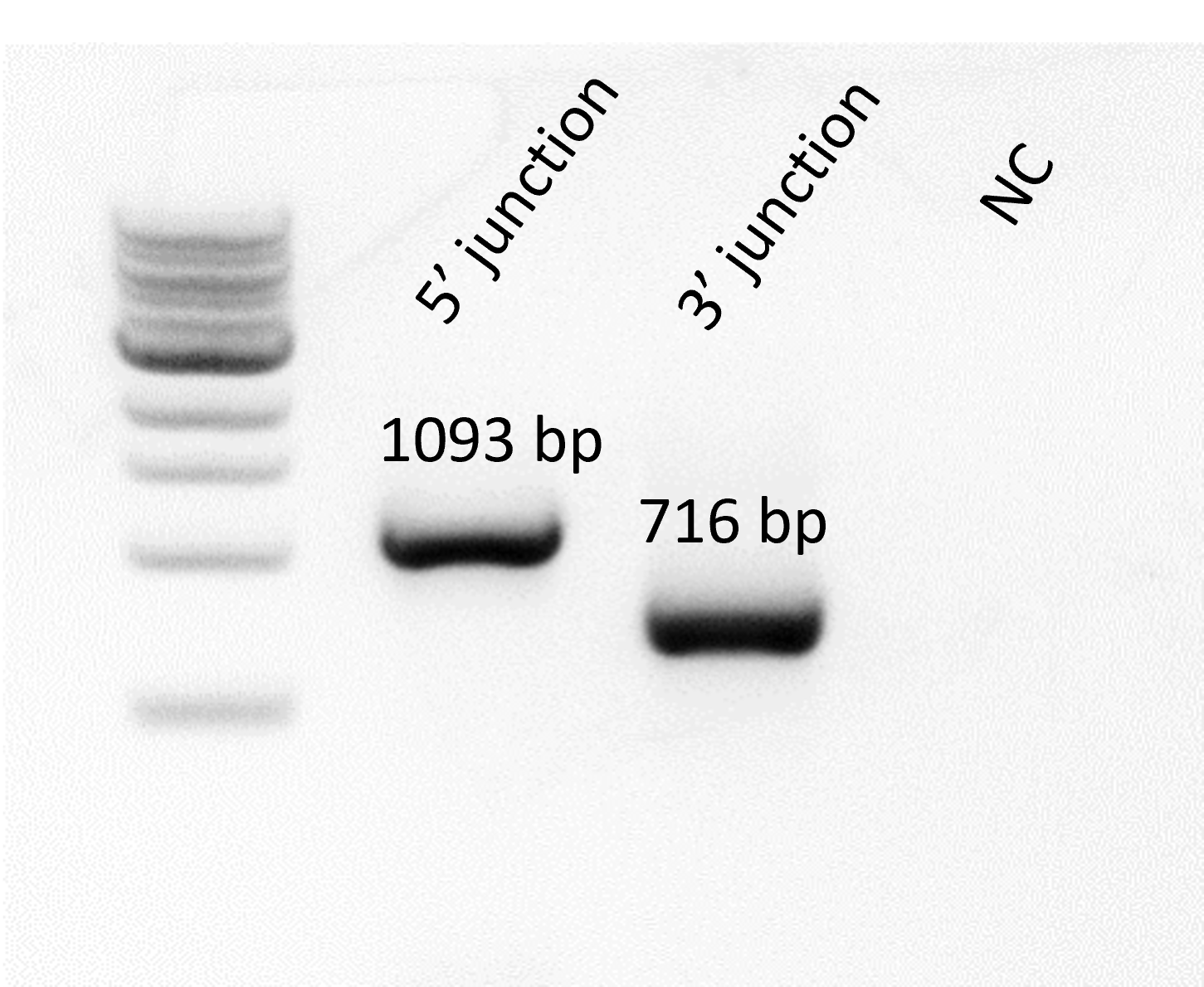

Supplement: Figure 2—figure supplement 1—source data 2. [file elife-105788-fig2-figsupp1-data2.zip › Figure 2-figure supplement 1-source data 2.tif]

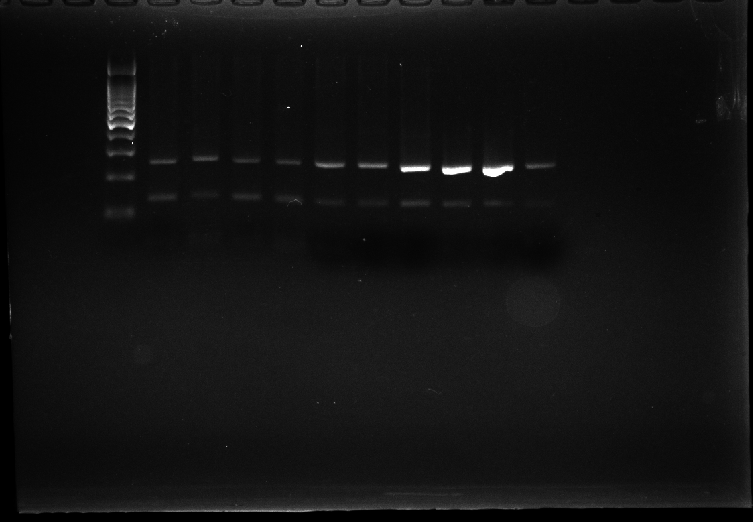

Supplement: Figure 6—source data 1. [file elife-105788-fig6-data1.zip › Figure 6-source data 1-1.tif]

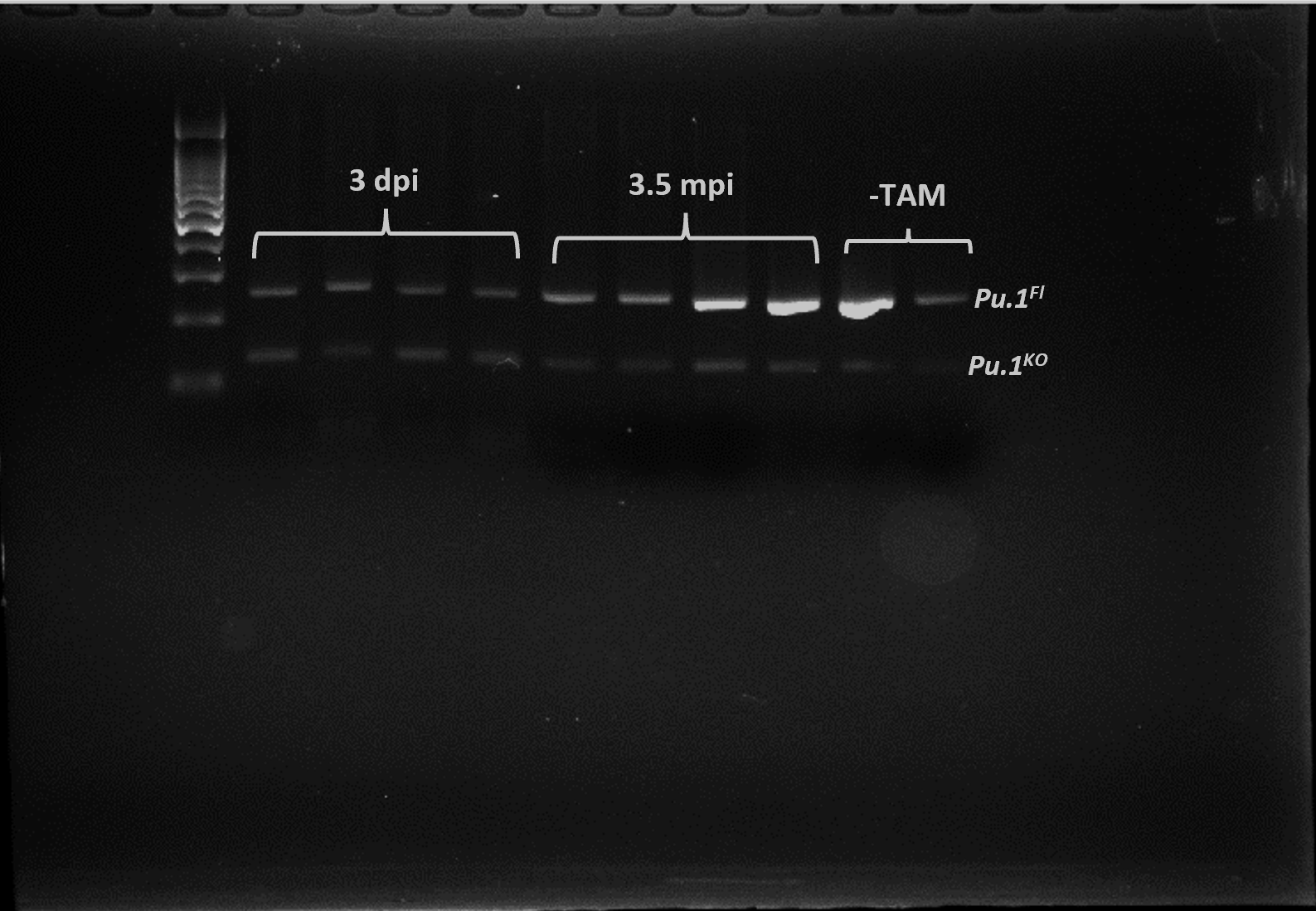

Supplement: Figure 6—source data 2. [file elife-105788-fig6-data2.zip › Figure 6-source data 1-2.tif]

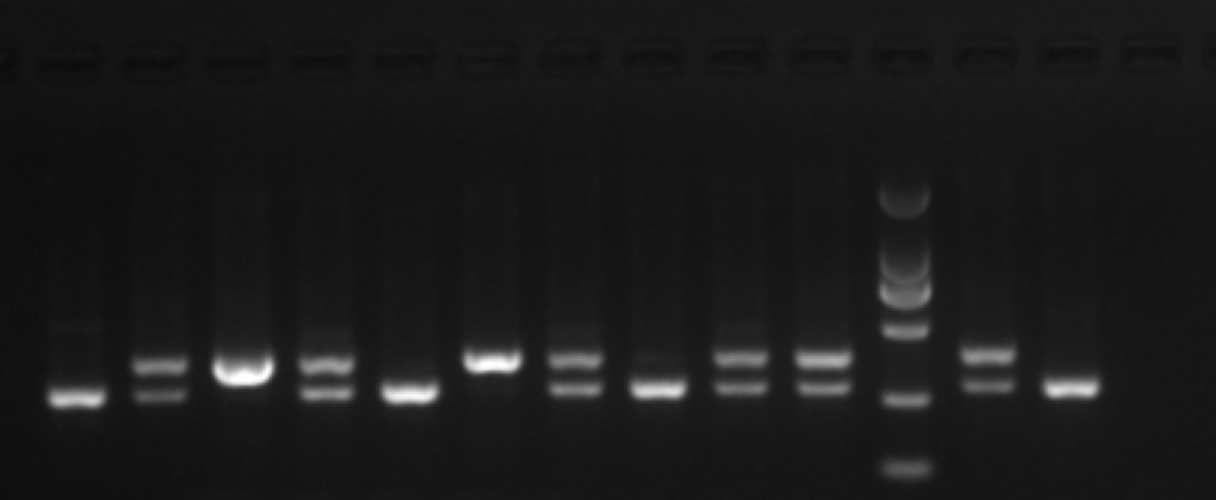

Supplement: Figure 6—figure supplement 1—source data 1. [file elife-105788-fig6-figsupp1-data1.zip › Figure 6-figure supplement 1-source data 1.tif]

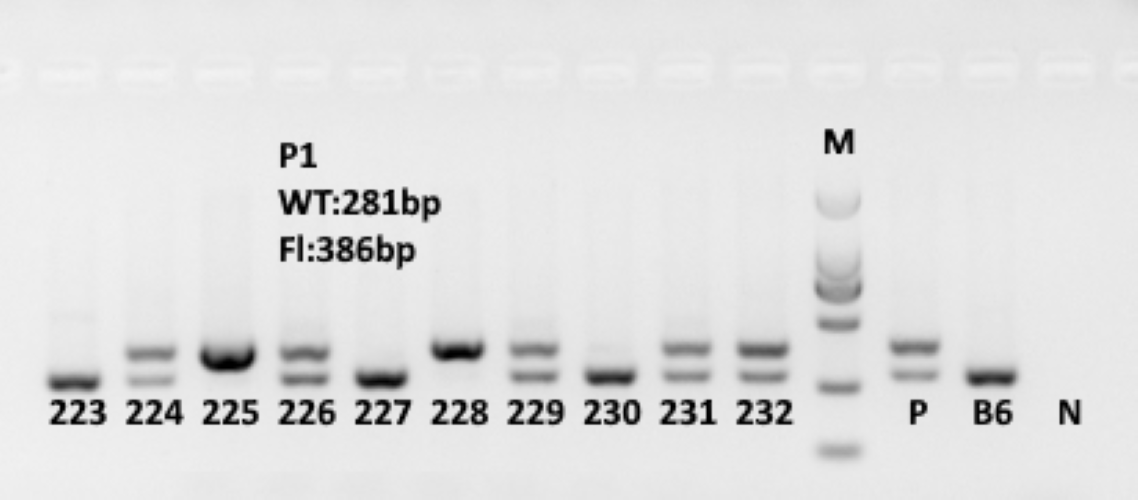

Supplement: Figure 6—figure supplement 1—source data 2. [file elife-105788-fig6-figsupp1-data2.zip › Figure 6-figure supplement 1-source data 2.tif]
